# Supplementary material for: Yellow fever in Ghana: Predicting emergence and ecology from historical outbreaks
Source: medRxiv. 2024 Jan 30:2024.01.29.24301911. Preprint. [Version 1] doi: 10.1101/2024.01.29.24301911 (PMC10862978; doi:10.1101/2024.01.29.24301911)
Supplement: Supplement 1 [file NIHPP2024.01.29.24301911v1-supplement-1.pdf]

medRxiv preprint doi: https://doi.org/10.1101/2024.01.29.24301911; this version posted January 30, 2024. The copyright holder for this preprint (which was not certified by peer review) is the author/funder, who has granted medRxiv a license to display the preprint in perpetuity.
It is made available under a CC-BY 4.0 International license .

Supporting Information

S1 Fig. Yellow Fever Annual Cases and Deaths in Ghana 1910-2022

The reported annual number of YF cases and deaths in Ghana since the first detected outbreak in 1910 until 2022 are shown. Also depicted are the years of reactive YF vaccination campaigns and immunization policies.
